# Comparison of qPCR and blood smear microscopy for the diagnosis of *Mycoplasma suis* in a French veterinary practice

**DOI:** 10.1186/s40813-019-0143-8

**Published:** 2020-02-20

**Authors:** Valérie Normand, Gwenaël Boulbria, Mathieu Brissonnier, Véronique Bachy, Pierre-Yves Moalic, Pauline Berton, Franck Bouchet, Arnaud Lebret

**Affiliations:** 1 Porc.Spective Swine Vet Practice, ZA de Gohélève, rue Joseph et Etienne Montgolfier, 56920 Noyal Pontivy, France; 2 Orbio Finalab Veterinary Laboratory Group, 12 rue du 35ème regiment d’aviation, 69500 Bron, France; 3 Labofarm Finalab Veterinary Laboratory Group, 4 rue Théodore Botrel, 22600 Loudéac, France

**Keywords:** Pig, *Mycoplasma suis*, PCR, Blood smear, Diagnostic

## Abstract

*Mycoplasma suis* (*M. suis*) is an haemotropic Mycoplasma that adheres and invades erythrocytes and is responsible for infectious anaemia of pigs. Infections with *M. suis* have been reported worldwide. Clinical signs after *M. suis* infection can be significant particularly for the breeding herd in the period around farrowing but consequences are highly variable with some infected pigs never exhibiting clinical disease. The study aimed to determine the clinical relevance of Giemsa-stained blood smear for the diagnosis of *M. suis* compared with qPCR results. In our study, the comparison of qPCR results with microscopic investigation of Giemsa-stained blood smears revealed a lower sensitivity of the microscopic method: only 33 out of 102 qPCR positive blood samples were microscopically positive (*M. suis* visualised). No relationship between mean qPCR loads and microscopic observation was observed. Although more costly, qPCR is probably the best diagnostic tool available today for *M. suis* diagnosis.

## Background

*Mycoplasma suis (M. suis)* is an uncultivable haemotropic Mycoplasma that targets red blood cells of pigs [3] and is responsible for infectious anaemia of pigs (IAP), historically known as porcine eperythrozoonosis [4]. The syndrome has two main clinical forms: an acute form with high fever and anaemia, and a chronic form with multiple and non-specific symptoms. Clinical consequences of *M. suis* infection can be very significant, particularly for the breeding herd in the period around farrowing. Infection with *M. suis* is also reported to result in decreased birth weights and poor growth in post-weaning piglets [4]. *M. suis* direct diagnosis consists in DNA detection by qPCR or observation in blood smears. To date, microscopic observation have proven to be of low sensitivity but it is of interest for practitioners because it could be performed immediately after clinical examination. A previous study described the low sensitivity of microscopic observation compared with qPCR, but the comparison was performed on post-weaning pigs (20–30 kg) supposed to be acutely diseased [7]. The objective of this study was to evaluate the clinical relevance of these two diagnostic tests for veterinary practitioners in adult pigs (sows) chronically affected.

## Materials and methods

A total of 199 sows from ten farms were individually sampled in the week before farrowing. Blood was collected by venipuncture (jugular vein). Two samples were collected in Vacutest® EDTA-anticoagulated tubes, one for qPCR and one for blood smears, and submitted to the diagnostic laboratory within 24 h under positive-cold conditions.

For qPCR, deoxyribonucleic acid (DNA) was extracted from 200 μL EDTA blood samples using MagAttract 96 Cador Pathogen kit (Qiagen, Venlo, The Netherlands) following manufacturer’s instructions. DNA recovery was obtained in 100 μL elution buffer AVE and stored at − 20 °C. A specific plasmid containing the targeted DNA sequence of *M. suis* was constructed. This plasmid was ordered (Eurofins, Luxembourg, Luxembourg): it contained the *M. suis* PCR target sequence. Dilutions of plasmidic DNA was then used to establish a quantitation curve. Dilutions were then used for absolute quantification assays. *M. suis* detection was achieved using a qPCR test [2]. The reverse primer, targeting 16S ribosomal DNA, was slightly modified. Following sequence alignment of French field strains of *M. suis*, reverse primer has been shifted from a base to the 3′ end. So that the test could be run with Labofarm’s routine qPCR thermal cycle, using the Ultra-Fast qPCR kit (Agilent Technologies, Les Ulis, France). Specificity was evaluated using *Mycoplasma hyopneumoniae*, *Mycoplasma hyorhinis*, *Mycoplasma hyosynoviae* and *Mycoplasma floculare* DNA. No cross reaction was detected. The quantification limit was achieved using *M. suis* negative EDTA blood sample spiked with *M. suis* plasmid. The qPCR is able to detect 10^6^ copies of 16S ribosomal DNA gene per ml of blood corresponding to 5 to 2.5 × 10^5^ bacteria per ml of blood.

**Fig. 1:**
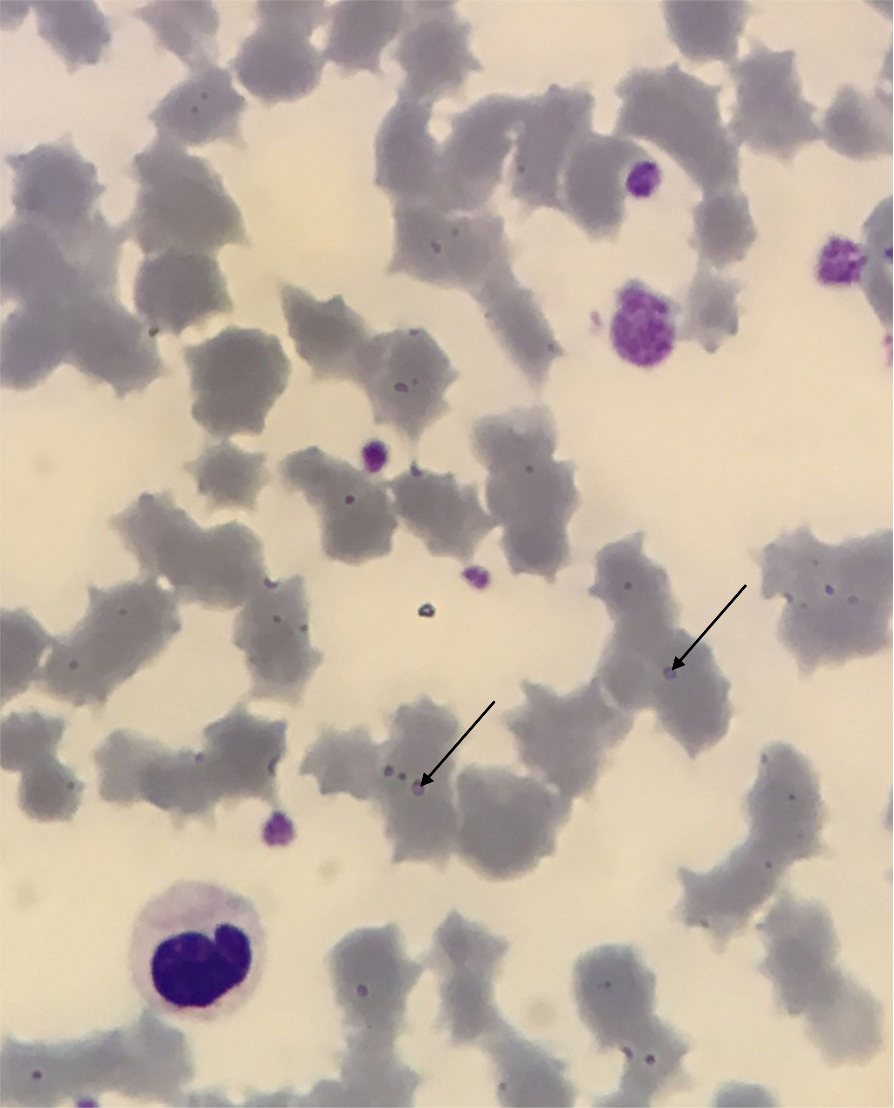
Light microscopic image of a Giemsa-Grünwald-stained infected blood smear. *M. suis* is identified with arrows (×1,000)

Giemsa-stained blood smears were prepared from EDTA-anticoagulated venous bloods using automatic Giemsa colouring with the Aerospray automaton (Elitech, Puteaux, France). They were then read on an Olympus CX41 microscope (Olympus, Tokyo, Japan) at × 1000 magnification (immersion oil). Smears were examined by trained haematopathologists. Blood smears were considered positive if the presence of *M. suis* was clearly identified (Fig. 1). Doubtful samples were blood smears on which no *Mycoplasma* sp. could not be directly visualised but where a cytopathogenic effect on erythrocytes was observed: mostly presence of ghost cells, polychromasia and anisocytosis. For the comparison between the two diagnostic tools, we considered that the blood smear was negative if no M. suis was observed.

Performances of qPCR and blood smear microscopy were compared to determine their clinical relevance. Specificity, sensitivity, positive predictive value (PPV) and negative predictive value (NPV) were estimated for Giemsa-stained blood smears using qPCR as the Gold Standard. The number of *M. suis* 16S gene copies per millilitre for each blood smear result (positive, doubtful or negative) were shown in box-plots using median, quartiles, minimum and maximum. Comparison of means was made using the Kruskal-Wallis test. Statistical significance was set at *p* < 0.05.

## Results

**Fig. 2:**
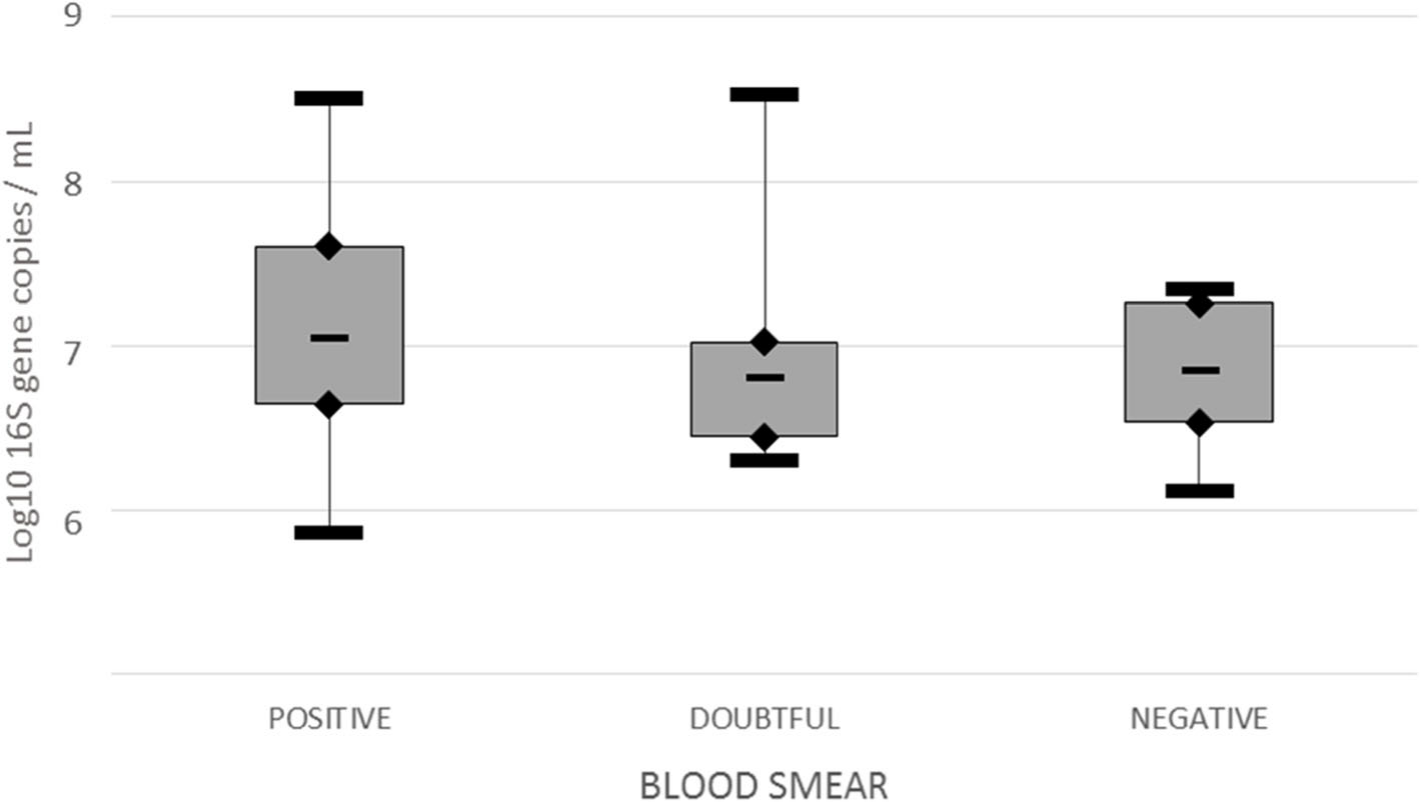
qPCR loads of *M. suis* positive, doubtful or negative sows based on blood smear microscopy. This graph is based on logarithmic scaling excluding qPCR unquantifiable results

Samples were collected from 199 sows, but comparison between qPCR and blood smears microscopic observation results could only be carried out on 191 samples because of unreadable blood smears. For the detection of *M. suis* with blood smears, 42 samples were positive, 41 were doubtful and 108 were negative. For qPCR, 102 samples were positive and 89 were negative. Doubtful microscopy observations, on which no *M. suis* could be visualised were considered as negative for the estimation of sensitivity, specificity, PPV and NPV (Table 1). Considering qPCR as the reference standard diagnostic tool for the detection of *M. suis*, we first determined a specificity of 90%, a sensitivity of 32%, a PPV of 79% and an NPV of 54% for the microscopic observation of Giemsa-stained blood smears. Excluding unquantifiable positive qPCR results, we found no statistical relationship (*p* > 0.05) between mean qPCR loads and blood smear results (Fig. 2). At farm level, two farms out of ten were incorrectly classified as negative based on blood smears (Table 2).

**Table 1:** Classification of qPCR and blood smear results

		qPCR		Total
		Positive	Negative	
Blood smear	Positive	33	9	42
	Negative	69	80	149
	Total	102	89	191

**Table 2:** Intra-herd rate of positive sows depending on diagnostic tool used (Giemsa-stained blood smear or qPCR)

Herd	Rate of positive sows based on Giemsa-stained blood smears results	Rate of positive sows based on qPCR results
A	22.2%	50.0%
B	57.9%	94.7%
C	26.3%	63.2%
D	15.0%	55.0%
E	10.5%	47.5%
F	20.0%	40.0%
G	21.1%	52.6%
H	0%	30.0%
I	0%	5.9%
J	40.0%	90.0%

## Discussion

The study aimed to determine the clinical relevance of Giemsa-stained blood smear for the diagnosis of *M. suis* compared with qPCR results. Indeed, qPCR is considered as the most effective method for the identification of *M. suis* [3]. In our study, we observed a low sensitivity of blood smear microscopy. Microscopic observation of the organism on the surface of erythrocytes in Giemsa-stained blood smears was demonstrated of poor relevance in acute infection ([3]; Hoelzle et al., 2007) because the parasites are not always apparent unless parasitaemia is present [6]. Chronic haemoplasma infections in pigs with low or undetectable numbers of parasites in peripheral blood smears are well recognized [5]. Limited number of *M. suis* on and between erythrocytes in the acute and chronic phases could explain the low sensitivity of blood smear microscopy compared with qPCR [1]. *M. suis* can invade erythrocytes [1], which explains why the bacteria is detected by qPCR, sometimes with high bacterial loads, without microscopic detection. In other words, qPCR is probably the best diagnostic tool available today for the diagnosis of *M. suis* in veterinary practice. However, nine samples were only positive in blood smears. Two explanations could be exposed. These unexpected results could be linked to qPCR inhibitors in samples or could be false positive results in blood smears microscopic observations. Indeed, false positive microscopic results are possible by confounding *M. suis* and immature DNA harbouring erythrocytic structures [7].

## Conclusion

This study demonstrated the low sensitivity and the poor clinical relevance of blood smear microscopy for the detection of *M. suis* in chronically affected sows. Although more costly, qPCR is probably the best diagnostic tool available today for *M. suis* diagnosis.

## Data Availability

All datasets used in this study are available from the corresponding author on reasonable request.
